# Cluster analysis of personality traits in psychiatric patients with borderline personality disorder

**DOI:** 10.1186/s40479-022-00178-w

**Published:** 2022-02-08

**Authors:** Kristin Oladottir, Martina Wolf-Arehult, Mia Ramklint, Martina Isaksson

**Affiliations:** 1 Department of Neuroscience, Psychiatry, Uppsala University, Entrance 10, Floor 3B, SE-751 85 Uppsala, Sweden; 2 Present address: Psychiatry Northwest, Region Stockholm, PO Box 98, SE-191 22 Sollentuna, Sweden

**Keywords:** Borderline personality disorder, Cluster analysis, Subtypes, Endophenotypes, Swedish universities scales of personality (SSP), Personality traits, Alternative model for personality disorders (AMPD), ICD-11

## Abstract

**Background:**

Though the heterogeneous expression of symptoms of borderline personality disorder (BPD) is well-known, it is far from fully understood. Hybrid models combining dimensional and categorical ways of diagnosing BPD have been suggested to better handle this heterogeneity, but more research is needed. The aim of this study was to identify potential clusters in BPD, and evaluate if these clusters differed in diagnostic composition, severity, psychiatric symptoms, emotion regulation and control, or sociodemographic features.

**Methods:**

Clusters were based on personality traits measured with the Swedish universities Scales of Personality (SSP) in 141 psychiatric patients diagnosed with BPD. Hierarchical cluster analysis was performed using Ward’s method. We used one-way analysis of variance to explore the different clusters’ properties. Effect sizes were calculated using partial eta squared.

**Results:**

We found three distinct clusters: the lower psychopathology cluster (*N* = 67), the externalizing cluster (*N* = 28), and the internalizing cluster (*N* = 46). The clusters differed regarding trait composition, severity, and emotion regulation and control.

**Conclusions:**

Our findings support hybrid models for diagnosing BPD by showing that clusters differed in terms of both severity (lower and higher psychopathology) and personality traits/style (internalizing and externalizing). Assessment of personality traits may be a feasible way to differentiate between clusters. In the future, this knowledge might be used to personalize treatment.

## Introduction

Borderline personality disorder (BPD) is a heterogeneous disorder [1]. A person needs to fulfill only five out of nine diagnostic criteria to get a diagnosis. Hence, there are 256 combinations that can result in the same BPD diagnosis and it is possible for two individuals with BPD to share only one criterion [2]. Patients with BPD are also highly overrepresented in terms of psychiatric comorbidity [1, 3], further contributing to the heterogenous display of the disorder. Treatments for BPD have been shown to be effective for externalizing symptoms such as suicidal, self-harming, and impulsive behaviors, but not as effective in reducing depressive symptoms, which are often present in more internalizing ways of coping [4, 5]. Difficulties in providing adequate help to patients with BPD could at least partially be a consequence of this heterogeneity.

Identifying subtypes is one way of trying to understand the heterogeneity of BPD. Cluster analyses have been performed to identify meaningful subgroups of the disorder. Several researchers have based their clusters on personality disorder ratings [2, 6–12]. Others have based their analyses on emotional regulation strategies [13], intrapersonal problem inventories [14, 15], suicidal behavior [16], or a variety of demographic and psychological variables [17]. To date, findings on subtype patterns of BPD are diverse [10] – with differing numbers of clusters comprising differing characteristics – and consensus is not achieved. However, there are patterns that have recurred to some extent. One of the most common is the presence of an internalizing/inhibited cluster characterized by depressed mood, fear, and avoidance, and an externalizing/disinhibited cluster related to disinhibition and impulsivity [2, 10, 11, 13, 14]. Another recurring characteristic is the presence of clusters that differ in symptom severity or function [2, 10, 11, 13]. However, it is important to highlight that some researchers have found other subtypes not resembling these categories [7, 12] and that the heterogeneity in BPD is far from resolved [10].

Subtyping in previous research has largely been based on the categorical way of diagnosing BPD, assuming that the diagnosis is either present or not. However, the categorical approach has been highly debated and criticized [18], with newer findings indicating that a dimensional approach would be more appropriate for diagnosing personality disorders [1]. After extensive research [19, 20], a hybrid model that includes two main components was suggested for the new version of the Diagnostic and Statistical Manual of Mental Disorders (DSM-5) [21]: 1) a *severity or impairment* criterion (dimensional) comprising four elements related to one’s sense of self and interpersonal relationships, and 2) a *style* criterion comprising 25 facets (categorical), divided into the following broad domains: negative affectivity, detachment, antagonism, disinhibition, and psychoticism. Due to insufficient research and consensus, the old categorical way of diagnosing BPD was retained in the DSM-5. However, the Alternative Model for Personality Disorders (AMPD), which included the new suggestions, was added into a separate section of the manual for further research. In contrast, a hybrid model similar to the AMPD was included in ICD-11 [22], replacing the categorical description, with a dimensional severity indicator (personality difficulty, mild personality disorder, moderate personality disorder, and severe personality disorder) and a categorical trait indicator (negative affectivity, detachment, dissociality, disinhibition, and anankastia).

Criticism of the new dimensional or hybrid approach has included, for example, discrepancies regarding which facets, styles, or traits that are strong enough to be included in the model [23], and results failing to support a hybrid model [1, 7]. Studies designed with a basis in cluster subgroups of BPD using the hybrid approach have been performed. For example, Gamache et al. [10] found four clusters based on the AMPD, comprising a dimensional component of severity (BPD traits, moderate pathology, and severe pathology), with the moderate pathology cluster separated into two categorical dimensions of impulsivity vs. identity/depressiveness, resembling the externalizing-internalizing findings of previous studies [2, 13].

In the present study, we aimed to identify clusters in BPD based on personality traits known to be related to vulnerability for developing psychopathology. We also wanted to evaluate if the clusters differed in diagnostic composition, severity, psychiatric symptoms, emotion regulation and control, or sociodemographic features. We hypothesized that we would find distinct clusters. Although our approach was exploratory, with the aim to evaluate clusters in terms of both content and number, we hypothesized that we would find clusters characterized by traits related to inhibition/internalization and disinhibition/externalization, with the inhibited cluster showing less adventure seeking, impulsiveness, and aggressiveness.

## Methods

### Participants

**Fig. 1:**
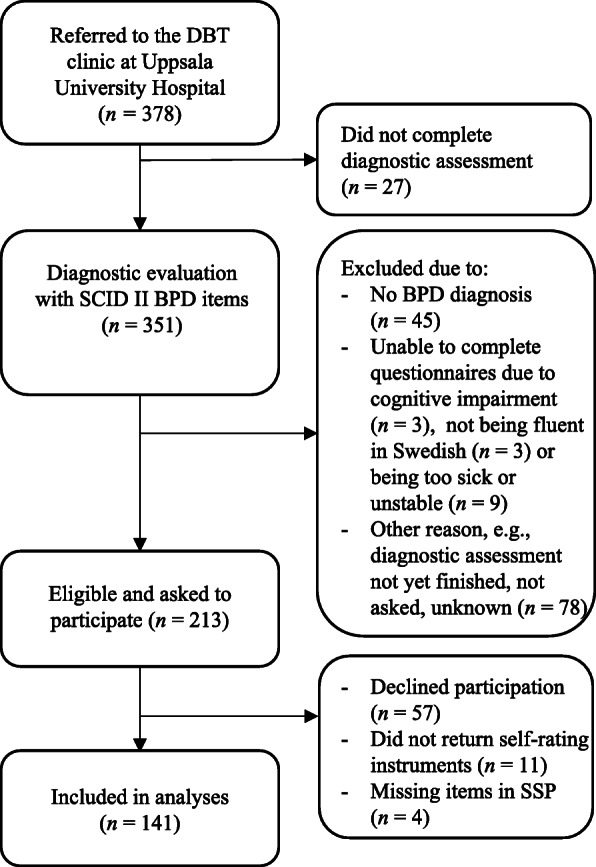
Flowchart of participant recruitment. *Note.* SCID-II = Structured Clinical Interview for axis II personality disorders, BPD = borderline personality disorder, DBT = dialectical behavior therapy, SSP = Swedish universities Scales of Personality

Participants were 141 psychiatric patients who completed a diagnostic evaluation with the Structured Clinical Interview for axis II personality disorders (SCID-II; 24) at the dialectical behavior therapy (DBT) clinic in Uppsala, Sweden. The clinic treats patients with BPD, and in some cases patients fulfilling four criteria (i.e., not fulfilling a BPD diagnosis). The inclusion criterion for this study was that a patient fulfilled the criteria for a full BPD diagnosis. Exclusion criteria were if the patient was unable to complete the questionnaires, for example, if the patient was too sick or unstable, if they had a cognitive impairment, or if they had insufficient knowledge of the Swedish language. A flowchart of the recruitment process is presented in Fig. 1; demographic characteristics are presented in Table 1.

**Table 1:** Demographic characteristics of the 141 participants diagnosed with borderline personality disorder

	***n*** (%)
**Civil status**	
In a relationship	79 (56.0)
Single	61 (43.3)
No information	1 (0.7)
**Gender**	
Male	16 (11.3)
Female	125 (88.7)
**Occupation**	
Working/studying	80 (56.7)
Not working/studying	60 (42.6)
No information	1 (0.7)
**Educational attainment**	
Primary school	32 (22.7)
Secondary school	79 (56.0)
Post-secondary education	29 (20.6)
No information	1 (0.7)

### Instruments

#### Swedish universities scales of personality

The Swedish universities Scales of Personality (SSP) is a self-rating scale that measures stable personality traits based on biological theories related to psychopathologies [24]. The forerunner, the Karolinska Scales of Personality, was developed in 1970 with the intention to quantify well-established personality constructs known to be related to psychiatric disorders. The scale underwent a thorough revision in 2000 to improve its psychometric qualities, and the revised version was called SSP [24]. SSP has 91 items that the patient answers on a Likert scale ranging from 1 to 4 (1 = not true at all, 4 = exactly right). SSP consists of 13 subscales divided into three overarching factors: 1) the Neuroticism factor, containing the subscales Somatic trait anxiety, Psychic trait anxiety, Stress susceptibility, Lack of assertiveness, and Embitterment, 2) the Aggressiveness factor, containing the subscales Social desirability (reversed), Trait irritability, Verbal trait aggression, and Physical trait aggression, and 3) the Extraversion factor, containing the subscales Impulsiveness, Adventure seeking, and Detachment (reversed). High scores indicate more distinct features of that trait. Results are presented as t-scores that are standardized in relation to the general population. The mean score is 50, with 10 being equivalent to one standard deviation. Internal consistency has been satisfactory for all subscales except social desirability [24]. SSP encompasses a range of questions measuring personality traits that are adaptive or not necessarily maladaptive (e.g., “I don’t have much patience”) and personality traits that are generally maladaptive (e.g., “If someone hits me, I hit back”). A comparison between SSP and several other personality instruments, including the revised NEO personality inventory (NEO-PI-R), related to the Five Factor Model of personality, was recently published [25]. Strong correlations were found between SSP and NEO-PI-R regarding neuroticism, extraversion, and aggression/agreeableness, while correlations between other scales were weaker (Fagerberg 2021). We chose SSP as our cluster instrument, as it is a well-validated instrument that encompasses traits shown to correlate with psychiatric psychopathologies. Since the new hybrid models in DSM-5 and ICD-11 aim to differentiate between personality traits/styles, information about clusters based on this instrument could contribute to future debate. In the present study, personality traits or groups of personality traits (factors) from SSP are comparable to personality styles/traits/facets as described in the AMPD and the ICD-11 model.

#### Borderline symptom list – short version

The Borderline Symptom List – short version (BSL-23) is a scale that estimates the severity of BPD symptoms [26, 27]. It includes 23 items, and is a condensed version of BSL-95 [28]. The psychometric properties of BSL-23 have been compared with BSL-95, showing a high correlation of mean scores between the scales. The score of BSL-23 is calculated as the mean of all items. Patients evaluate the symptoms they have had during the preceding week on a Likert scale ranging from 0 to 4 (0 = no symptoms, 4 = very strong). BSL-23 has good test-retest reliability, excellent internal consistency, and good validity [26]. The Swedish version has not yet been evaluated.

#### Difficulties in emotion regulation scale – short version

The Difficulties in Emotion Regulation Scale – short version (DERS-16) [29] is a condensed version of the self-rating scale DERS [30] that estimates different dimensions of difficulties in emotion regulation. DERS-16 contains 16 items that are rated on a Likert scale ranging from 1 to 5 (1 = almost never, 5 = almost always). Higher scores on DERS-16 reflect more difficulties in emotion regulation. DERS-16 has good test-retest reliability, excellent internal consistency, and has been validated in Swedish [29].

#### Ego Undercontrol scale – short version

The Ego Undercontrol Scale – short version (EUC-13) is a self-rating scale that estimates emotion control or, in other words, the ability to inhibit emotions and expression in order to pursue long-term goals [31]. The scale provides an estimate of where an individual rates themself on the spectrum from high emotion control (overcontrol) to low emotion control (undercontrol). Overcontrolled individuals are inhibited in affection and expressiveness. Undercontrolled individuals express strong affects and are impulsive. The patient responds on a Likert scale from 1 to 4 (1 = not at all, 4 = totally agree). EUC-13 has good test-retest reliability. The internal consistency is satisfactory, except for the questions about socially restrained behavior [31]. The global score is recommended for assessing over-/undercontrol. The scale is based on a longer version with 37 items [32]. In the beginning of the study, the condensed-13 item version was not yet finalized. Therefore, the 13 items were extracted from the longer version in this study. EUC-13 has been validated in Swedish [31].

#### Hopkins symptom checklist – short version

The Hopkins Symptom Checklist – short version (HSCL-25) is a condensed version of HSCL [33], which is a self-rating scale screening for symptoms of anxiety and depression. If the mean rating is equal to or higher than 1.75, it indicates that the patient is in need of treatment [34]. HSCL-25 contains 25 items which the patient answers on a Likert scale ranging from 1 to 4 (1 = not at all, 4 = very). HSCL-25 has satisfactory reliability and validity, and has been validated in Swedish [34].

#### Structured clinical interview for axis II personality disorders

SCID-II is a semi-structured clinical interview [35] used to diagnose personality disorders in accordance with the DSM [21, 36]. It is a well-studied, reliable, and valid instrument considered appropriate for diagnosing personality disorders, including BPD [37]. The evaluation of BPD includes nine items, each representing a diagnostic criterion. During the interview, participants are asked a number of questions about each item so the interviewer can evaluate whether the criterion is not fulfilled, partly fulfilled, or fulfilled. The SCID-II has shown good intra-rater and inter-rater reliability [38]. For the present study, the general personality disorder criteria and the borderline items were used.

The staff in the DBT team performing the interviews were five experienced psychologists. One of the authors (MWA), who was team leader at the time, was seen as an expert and initially co-rated one filmed interview from each psychologist. She agreed with the evaluation in all cases. Later in the study, seven new raters were trained before being included as interviewers. They showed complete agreement regarding current BPD diagnosis in four out of five interviews (80%), with prevalence and bias-adjusted kappa [39] ranging from 0.6 to 1.0 between assessors.

### Missing data

Missing data were rare. For SSP, missing values were handled using a syntax where, if one item for one subscale was missing, the scale was divided by six instead of seven. If more than one item was missing, the whole scale was deleted from the analyses; the participant was then also removed from the analyses. Missing values for the other instruments were: < 1% for BSL-23, DERS-16, and HSCL-25, and 2.3% for EUC-13. Missing values were handled through mean imputation, meaning that the missing item was replaced by the item average for that subscale.

### Data analysis

**Fig. 2:**
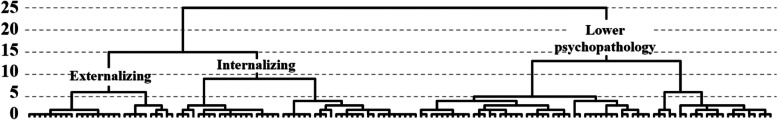
Dendrogram showing hierarchical cluster analysis in accordance with Ward’s method. *Note.* Euclidian distances were used to find clusters in borderline personality disorder. Each leaf on the horizontal axis represents a participant. The vertical axis represents the distance between the clusters

To find out if there were different subgroups of BPD, we used Ward’s hierarchical cluster analysis with squared Euclidean distances. Cluster memberships were tested for two- to six-cluster solutions on the 13 SSP subscales (see dendogram in Fig. 2). Six was set as the maximum, as a larger number of clusters would yield too small groups (a maximum of 20 cluster members, if distributed equally across seven clusters). Hierarchical cluster analysis is exploratory, and the optimal solution is chosen based on reviewing the dendrogram, and evaluating whether the clusters in different solutions yield meaningful differences with regard to the outcomes. To explore the different cluster properties, we used one-way analysis of variance (ANOVA) for the following scales: SSP, HSCL-25, BSL-23, EUC-13, and DERS-16. Tukey’s Honest Significant Difference was used as a post-hoc test. Effect sizes were calculated using partial eta squared (partial η^2^). For nominal data, we used the chi-squared test and Bonferroni’s post-hoc test, with Cramér’s V for calculations of effect sizes. The data were processed with SPSS version 26 for Windows. The significance level was set to 5%.

### Ethical considerations

Ethical approval was granted by the Regional Ethics Committee in Uppsala, ref. no. 2013/156 and 2014/252. All participants gave informed consent.

## Results

### Cluster analysis

The three-cluster solution was superior to the others since it demonstrated distinct psychopathology between the clusters, differentiating them from each other. The two-cluster solution provided two distinct clusters (one lower and one higher psychopathology), but failed to capture the meaningful differences, supported by the ANOVA, when the higher psychopathology cluster was separated into two. Solutions with four clusters or more did not separate the clusters as well as the solutions with two or three clusters. The clusters in the chosen three-cluster solution were named the lower psychopathology cluster, the externalizing cluster, and the internalizing cluster. The three clusters were similar with respect to civil status, educational attainment, gender, and age (See Table 2). Individuals in the internalizing cluster were more often on long-term sick leave or unemployed (57.8%) than those in the externalizing cluster (39.3%) or the lower psychopathology cluster (34.3%), see Table 2. The three clusters had a handful of features in common, but differed in regard to other characteristics, such as aggression and adventure seeking (details are presented below and in Table 3).

**Table 2:** Distribution of age, civil status, occupation, and education attainment for the BPD clusters

	Lower psychopathology cluster, ***n*** (%)	Externalizing cluster, ***n*** (%)	Internalizing cluster, ***n*** (%)	Post-hoc
**Civil status**^**1**^				
Single	31 (46.3%)	13 (46.4%)	17 (37.8%)	
In a relationship	36 (53.7%)	15 (53%)	28 (62.2%)	
**Occupation**^**2**^				
Working/studying	44 (65.7%)	17 (60.7%)	19 (42.2%)	C < A*
Not working/studying	23 (34.3%)	11 (39.3%)	26 (57.8%)	A < C*
**Educational attainment**^**3**^				
Primary school	19 (28.4%)	3 (10.7%)	11 (25.6%)	
Secondary school	32 (47.8%)	19 (67.9%)	28 (65.1%)	
Post-secondary education	16 (23.9%)	6 (21.4%)	4 (9.3%)	
**Gender**				
Female	58 (86.6%)	25 (89.3%)	42 (91.3%)	
Male	9 (13.4%)	3 (10.7%)	4 (8.7%)	
**Mean age (SD)**	26.8 (8.3)	26.6 (6.4)	26.7 (5.9)	

*Note.* *p < 0.05, ^1^ *n* = 140, ^2^ *n* = 140 ^3^, *n* = 138. Bonferroni’s post-hoc test was used for group comparisons

**Table 3:** Personality dimensions measured with SSP in the three-cluster solution of borderline personality disorder

	Lower psychopathology cluster, mean (SD)	Externalizing cluster, mean (SD)	Internalizing cluster, mean (SD)	Effect sizepartial η^**2**^	F	Post-hoc
**Somatic trait anxiety**	65.7 (8.9)	69.7 (7.5)	71.3 (9.0)	0.08	6.18	A < C**
**Psychic trait anxiety**	65.0 (8.4)	66.7 (8.7)	72.7 (5.9)	0.17	14.09	B < C**, A < C***
**Stress susceptibility**	71.0 (10.7)	70.0 (14.7)	79.6 (6.4)	0.14	11.23	B < C***, A < C***
**Lack of assertiveness**	56.9 (12.1)	49.9(8.0)	64.3 (10.4)	0.19	15.74	B < A**, A < C***, B < C*
**Impulsiveness**	59.5 (10.6)	66.5 (10.1)	56.2 (11.7)	0.10	7.86	A < B*, C < B***
**Adventure seeking**	49.7 (11.1)	56.9 (11.4)	42.9 (10.4)	0.17	14.57	A < B**, C < B***, C < A**
**Detachment**	50.3 (9.4)	56.2 (9.1)	62.4 (10.5)	0.24	21.58	A < B*, B < C*, A < C***
**Social desirability**	45.4 (11.4)	35.7 (9.3)	36.7 (11.7)	0.15	11.91	B < A***, C < A***
**Embitterment**	70.6 (9.5)	78.4 (7.0)	80.2 (9.0)	0.21	18.20	A < B***, A < C***
**Trait irritability**	61.9 (9.6)	73.5 (7.7)	70.0 (8.6)	0.23	20.90	A < B***, A < C***
**Mistrust**	64.3 (12.2)	80.1 (5.4)	78.1 (9.7)	0.34	34.96	A < B***, A < C***
**Verbal trait aggression**	54.4 (11.3)	76.2 (5.9)	60.9 (8.0)	0.43	52.52	A < B***, C < B***, A < C**
**Physical trait aggression**	51 (12.7)	73.6 (9.3)	59.4 (11.8)	0.35	36.65	A < B***, C < B***, A < C**

*Note*. Effect size partial η^2^ small: 0.01, moderate: 0.06, large: 0.14 (37). Post-hoc Tukey * *p* > 0.05, ** *p* < 0.01, *** *p* < 0.001. SSP = Swedish universities Scale of Personality

### BPD clusters in SSP

**Fig. 3:**
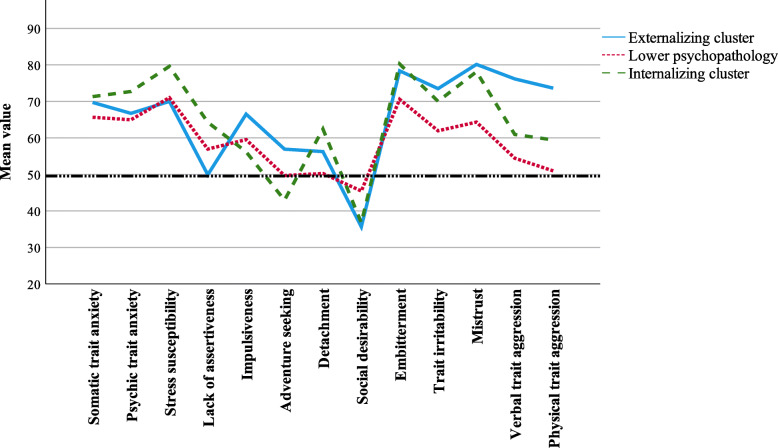
Personality dimensions for the lower psychopathology, the externalizing, and the internalizing clusters of borderline personality disorder. *Note.* Standardizing scores to the general population, 50 is the mean value of the population and 10 is one standard deviation. SSP = Swedish universities Scales of Personality

*The lower psychopathology cluster* (*N* = 67) demonstrated lower detachment, embitterment, irritability, mistrust, verbal aggression, and physical aggression than both the other clusters. It also displayed higher social desirability than the other clusters. *The externalizing cluster* (*N* = 28) demonstrated higher impulsiveness, adventure seeking, verbal aggression, and physical aggression than the other clusters. *The internalizing cluster* (*N* = 46) showed higher psychic anxiety, stress susceptibility, lack of assertiveness and detachment, and lower adventure seeking than the other clusters. Effect sizes between clusters were moderate to large.Output on SSP subscales from each cluster is presented in Fig. 3.

### Cluster differences in fulfilled SCID-II criteria

Difficulty controlling anger was the only diagnostic criterion that differed between the clusters (see Table 4), with the individuals in the externalizing cluster more often fulfilling the criterion than those in the lower psychopathology and internalizing clusters. The lower psychopathology cluster fulfilled fewer criteria than the externalizing and internalizing clusters.

**Table 4:** Fulfilled borderline personality disorder (BPD) diagnostic criteria for the three-cluster solution of BPD

	Lower psychopathology cluster, n (%)	Externalizing cluster, n (%)	Internalizing cluster, n (%)	Cramér’s V	Post-hoc
**1. Fear of abandonment**	36 (53.7%)	22 (78.6%)	26 (56.4%)	0.19	
**2. Unstable relationships**	53 (79.1%)	26 (92.9%)	36 (78.3%)	0.15	
**3. Identity disturbance**	30 (44.8%)	16 (57.1%)	23(50.0%)	0.09	
**4. Impulsiveness**	54 (80.6%)	22 (78.6%)	36 (78.3%)	0.07	
**5. Suicidal behavior**	57 (85.1%)	23(82.1%)	39 (84.8%)	0.03	
**6. Affective instability**	66 (98.5%)	27 (96.4%)	46 (100.0%)	0.11	
**7. Feeling of emptiness**	54 (80.6%)	24 (85.0%)	43 (93.5%)	0.26	
**8. Difficulty controlling anger**	45(67.2%)	27 (96.4%)	39(84.8%)	0.29	A < B**
**9. Dissociative symptoms, stress-related paranoia**	39 (58.2%)	18 (64.3%)	35 (76.1%)	0.13	
**Number of fulfilled criteria (SD)**	6.4 (1.2)	7.3 (1.2)	7.0 (1.1)		

*Note.* **p < 0.01. One item of missing data on impulsiveness. Two items of missing data on dissociative symptoms and stress-related paranoia. Estimated with mean (standard deviation) on number of fulfilled criteria. Effect size was calculated with Cramér’s V. Bonferroni was used for post-hoc analyses

### Cluster differences in BPD severity, emotion regulation and control, and overall psychiatric symptoms

As presented in Table 5, the one-way ANOVA showed that the lower psychopathology cluster reported less severe BPD symptoms (BSL-23 scores) and less difficulties in emotion regulation (DERS-16 scores) than the externalizing and internalizing clusters. In addition, the externalizing cluster was more undercontrolled, as assessed with EUC-13, than the internalizing cluster. The lower psychopathology cluster had lower total anxiety and depression, measured with HSCL-25, than the externalizing and internalizing clusters. However, all three clusters had depression and anxiety ratings higher than the clinical cut-off of 1.7 [33]. Effect sizes were generally moderate.

**Table 5:** Differences in BPD symptoms, emotion regulation, anxiety and depression between BPD clusters

		Lower psychopathology cluster, mean (SD)	Externalizing cluster, mean (SD)	Internalizing cluster, mean (SD)	Effect size partial η^**2**^	F	Post-hoc
**BSL**		2.2 (0.7)	2.7 (0.7)	2.7 (0.7)	0.12	9.35	A < B**, A < C***
**DERS-16**	Nonacceptance	3.4 (1.1)	3.6 (1.2)	3.9 (1.1)	0.12	2.12	
	Goals	4.2 (0.8)	4.4 (0.9)	4.6 (0.5)	0.08	5.55	A < C**
	Impulse	3.6 (1.1)	4.3 (0.9)	4.3 (0.8)	0.13	9.81	A < B**, A < C***
	Strategies	3.6 (1)	4.0 (0.9)	4.3 (0.6)	0.11	8.10	A < C***
	Clarity	3.5 (1)	3.9 (0.9)	3.9 (0.9)	0.05	3.40	
	Total	3.7 (0.7)	4.1 (0.7)	4.2 (0.6)	0.13	10.10	A < B*, A < C***
**EUC-13**		2.7 (0.5)	3.0 (0.4)	2.6 (0.5)	0.09	6.38	C < B*
**HSCL-25**	Anxiety	2.5 (0.5)	2.8 (0.5)	2.8 (0.5)	0.06	4.13	A < C*
	Depression	2.7 (0.6)	3.0 (0.5)	3.0 (0.5)	0.07	5.32	A < C**
	Total	2.6 (0.5)	2.9 (0.5)	2.9 (0.4)	0.08	5.93	A < B*, A < C**

*Note.* BPD = Borderline personality disorder. BSL-23 = Borderline Symptom List 23. DERS-16 = Difficulties in Emotion Regulation Scale-16. EUC-13 = Ego Undercontrol Scale – 13. HSCL-25 **=** Hopkins Symptom Checklist – 25. Three missing participants for DERS-16, three missing participants for EUC-13. Effect size partial η^2^ small: 0.01, moderate: 0.06, large: 0.14 (37). Post-hoc Tukey *p < 0.05, ** *p* < 0.01, *** *p* < 0.001

## Discussion

The current study aimed to identify possible clusters of BPD based on personality traits and to evaluate if these clusters differed in terms of psychiatric symptoms, severity, emotion regulation and control, or sociodemographic features. Three clusters were identified: the lower psychopathology, the externalizing, and the internalizing. The clusters differed from each other in terms of both severity (lower and higher psychopathology) and style (internalizing and externalizing). The latter supported our hypotheses. The clusters also shared many similarities, indicating a common psychiatric profile in BPD.

A three-cluster solution was determined as the best-fitting solution in this study, in line with several other studies [2, 11, 12, 17]. Previously, other cluster solutions have also been found. For example, Leihener et al. [14] and Soloff and Chiappetta [16] found two-cluster solutions, Gamache et al. [10] and Sleuwaegen et al. [13] found four-cluster solutions, while Salzer et al. [15] found a five-cluster solution.

One of the key findings in our study was the identification of a cluster that differed from the others in terms of severity. The *lower psychopathology cluster* was characterized by less detachment, embitterment, mistrust, irritability, verbal aggression and physical aggression, and more social desirability than the other clusters. Also, participants in this cluster had fewer problems with emotion regulation, less severe BPD symptoms and anxiety and depressive symptoms, and fulfilled fewer BPD criteria. Gustavsson et al. [24] have evaluated personality traits from SSP in a general, non-clinical, Swedish population, providing normative data that can be used for comparison in other studies. When contrasted to these non-clinical data, the lower psychopathology cluster was more similar to the ratings of a general population, with detachment, aggression, and social desirability closer to the population mean. Like some previous cluster analytic studies on BPD, both those based on categorical diagnostics and those based on hybrid/dimensional diagnostics, our findings supported the idea that a severity component is important for understanding the heterogeneity of the disorder [2, 10, 11, 13, 17]. For example, Smits et al. found a core BPD cluster characterized by more severe BPD [2]. Gamache et al. [10], who used the AMPD for diagnosing the sample (including subclinical BPD), found three levels of severity. Interestingly, when testing the different cluster solutions in this study, the two-cluster solution maintained the higher and lower psychopathology clusters, while the internalizing and externalizing clusters were merged – indicating severity as a distinct factor, separate from the qualitative differences between the two qualitative styles.

Another key finding was that the externalizing and internalizing clusters differed from each other, and that these differences were characterized by qualitative style differences, rather than severity. The *externalizing cluster* was characterized by traits like higher impulsiveness, adventure seeking, verbal aggression and physical aggression compared with both the other clusters. This group also showed higher undercontrol than the internalizing cluster. Several measures indicating severity, such as borderline severity symptoms, number of BPD criteria, difficulties regulating emotions, and anxiety and depression, differed only in contrast to the lower psychopathology cluster, not the internalizing cluster. Males were not overrepresented in the externalizing cluster, unlike what was seen in one earlier study with a similar cluster solution [2]. A similar externalizing cluster has been identified in several other studies [2, 10, 17]. Further, the *internalizing cluster* was characterized by greater psychic anxiety, stress susceptibility, lack of assertiveness and detachment, and lower adventure seeking than the other two clusters, as well as less undercontrol than the externalizing cluster. This cluster resembles clusters from prior studies, such as in Smits’ and coworkers’ [2] three-cluster solution, where both the core BPD cluster and the paranoid/schizotypal clusters showed traits representing avoidance. Similar clusters have been identified by for example Sluwaegen et al. [13], who found an inhibited cluster in their three-cluster solution, Gamache et al., who found a cluster on identity problems/depressiveness [10], and Digre et al. [17], who found two internalizing clusters with differing severities. The internalizing cluster has been presented under various names and in differing ways, depending on what outcome measures have been chosen, but is almost always characterized by some kind of inhibition and avoidance problems, whereas the externalizing cluster is characterized by externalization/disinhibition [2, 10]. Returning to the question of severity; even if most severity measures did not differ between the internalizing and externalizing clusters, some did. This is in line with previous research, where the internalizing subgroup has generally represented the most severe cluster with lower functioning and less response to treatment [17]. In our data, the internalizing cluster had a higher frequency of unemployment or sick leave, possibly suggesting greater work-related impairment. Interestingly, the internalizing cluster also represented the most neurotic cluster in terms of personality traits. This conclusion is based on scores on the neuroticism factor from SSP, comprising the subscales somatic trait anxiety, psychic trait anxiety, stress susceptibility, lack of assertiveness, and embitterment. On all these subscales, the internalizing cluster had the highest scores of the three clusters. Thus, one alternative interpretation of the clusters in our study could be a three-cluster solution where all three clusters represented different severities, with two also representing one of two styles: a lower psychopathology cluster, an extraverted-intermediate psychopathology cluster, and an introverted-severe psychopathology cluster. However, the similar ratings on anxiety/depression and borderline severity somewhat contradicted such an interpretation, which should therefore primarily serve as a hypothesis for future studies. The discrepancy between higher neuroticism, but similar levels of anxiety and depression and borderline severity, is interesting, and might be explained by differences in the concepts of trait and state. Specifically, a trait measure such as SSP might be valid for assessing personality, even in the presence of a comorbid psychiatric disorder (state) such as anxiety or depression. However, more studies are needed to explore the influence that state has on trait measures, since previous studies are not entirely consistent [25, 40].

It is important to mention that contrasting our cluster solution to previous studies is complex, since subtypes are often based on different measures, and different names are used for similar concepts. While this study is based on the subtypes on personality traits, others have based their subtypes on personality disorder assessment [2, 6–12], emotional regulation strategies [13], intrapersonal problem inventories [14, 15], suicidal behavior [16], or other psychological variables [17]. Replicating findings based on another perspective/measure can be a weakness, but also a strength. To our knowledge, clusters are rarely based on personality traits, even though traits are considered the inherited vulnerability in the main models for BPD development [1], meaning that this study makes an important contribution.

### Implications and future research

Critics might argue that subtyping of BPD has already been done and that the new AMPD approach should be used if new subtyping is done. However, the categorical approach remains the current choice for diagnosing BPD, the debate on which facets/styles that are the most salient for BPD is still highly current, and BPD heterogeneity has not been resolved [1]. To our knowledge, no personality measure specifically developed to identify personality traits related to development of psychopathology has previously been used when clustering BPD. This might add information to the debate on facets/styles that are important for inclusion in future models – either categorical, hybrid, or dimensional. This is supported by findings in a study by Mulay et al. [41], where substantial common ground was identified between these models, and where traditional categorical BPD criteria were translatable to the AMPD dimensional metric. Although our study was not based on the AMPD or ICD-II, it is interesting to see similarities with the results of the study by Gamache et al. [10], suggesting a severity component and two main facets/styles that seem especially important when diagnosing BPD. Neither the severity component nor the style component is included in the categorical way of diagnosing BPD [21]. Thus, our findings support hybrid models, as in ICD-11 [22], where these factors are taken into account. An instrument or clinician rating on severity/impairment, as well as on pathological traits, especially in terms of externalization versus internalization, could be added to the assessment procedure for diagnosing BPD. This study did not provide findings on which measures are appropriate, but this should be a goal for future studies. A second implication was the one on treatment, where differences in style and severity might raise the question of whether different interventions are needed for different patients. It has been suggested that the dimensional severity indicator might guide prognosis and intensity of treatment, while the traits/styles/facets could indicate the appropriate type of treatment [42]. Findings in this study do not provide any information on what treatment works for what style or severity; however, we have reasoned that the internalizing/depressive cluster, often characterized by less improvement in treatment [4, 5], could benefit from more extensive treatment. This group of patients might be in need of additional or different treatment interventions compared with other patients. While DBT is one of the most well-studied treatments for BPD [4], Radically Open DBT (RO DBT), an adaptation of DBT developed specifically for internalizing disorders/disorders of overcontrol, could be an option [43]. Another suggestion could be to add RO DBT skills training to standard DBT for this group in future studies.

### Limitations

The study has some limitations. First, even though the sample is as large as in previous cluster analyses of BPD [13, 14], the sample may be somewhat small to detect all potentially meaningful differences. Second, more females than males participated in this study, though males and females seem to have BPD to the same extent in the community [44], limiting the generalizability of our findings. Third, participants were recruited from a specialized DBT unit, thus probably representing a more severe subgroup of the BPD population, in need of full-scale specialized treatment that is not available at other clinics. Fourth, there are limitations with self-rating scales, for example, that people tend to answer the questions in a way that is favorable for them [45]. Adding a clinically rated instrument would have strengthened the findings. Lastly, this study lacks measures or ratings that are specifically developed to correspond to the AMPD/ICD-11 models for diagnosing BPD.

## Conclusions

In the present study, we identified three clusters of BPD: the lower psychopathology, the externalizing, and the internalizing. The three-cluster solution is the one most commonly identified, and this study has added further evidence to these findings. The small differences in diagnostic criteria found in our results, in combination with significant differences on other measures such as personality, emotion regulation and control, and severity, suggest that the current way of diagnosing BPD in DSM is inadequate. The three clusters showed similarities with several clusters identified in previous research – including a severity indicator, as well as an indicator of style (externalizing and internalizing), supporting hybrid models for diagnosing BPD. This knowledge might help us improve assessment and personalize treatment so that it better fits the needs of each specific BPD patient.

## Data Availability

Data will not be made publicly available due to confidentiality, but can be made available upon reasonable request to the corresponding author.

## Abbreviations

AMPD: Alternative Model of Personality Disorders
ANOVA: One-way analysis of variance
BPD: Borderline personality disorder
BSL-23: Borderline Symptom List – short version
DBT: Dialectical behavior therapy
DERS-16: Difficulties in Emotion Regulation Scale – short version
DSM-IV: Diagnostic and Statistical Manual of Mental Disorders
EUC-13: Ego Undercontrol Scale – short version
HSCL-25: Hopkins Symptom Checklist – short version
ICD: International Classifications of Diseases
RO-DBT: Radically Open Dialectical Behavior Therapy
SCID-II: Structured Clinical Interview for axis II personality disorders
SSP: Swedish universities Scales of Personality
